# An Automated Optical Strain Measurement System for Estimating Polymer Degradation under Fatigue Testing

**DOI:** 10.3390/s22166034

**Published:** 2022-08-12

**Authors:** Alexey A. Bogdanov, Sergey V. Panin, Pavel S. Lyubutin, Alexander V. Eremin, Dmitry G. Buslovich, Anton V. Byakov

**Affiliations:** 1 Laboratory of Mechanics of Polymer Composite Materials, Institute of Strength Physics and Materials Science of Siberian Branch of Russian Academy of Sciences, Tomsk 634055, Russia; 2 Department of Materials Science, Engineering School of Advanced Manufacturing Technologies, National Research Tomsk Polytechnic University, Tomsk 634050, Russia

**Keywords:** digital image correlation method, hysteresis loop, polymer fatigue, polyimide, cyclic load, mechanical properties

## Abstract

(1) Background: this study deals with design of an automated laboratory facility based on a servo-hydraulic testing machine for estimating parameters of mechanical hysteresis loops by means of the digital image correlation (DIC) method. (2) Methods: the paper presents a description of the testing facility, describes the grounds for calculating the elastic modulus, the offset yield strength (OYS) and the parameters of the mechanical hysteresis loops by the DIC method. (3) Results: the developed hardware-software facility was tested by studying the fatigue process in neat polyimide (PI) under various amplitude tension-tension loadings. It was found that the damage accumulation was accompanied by the decrease in the loop areas, while failure occurred when it reduced by at least ~5 kJ/m^3^. (4) Conclusions: it was shown that lowering the loop area along with changing the secant modulus value makes it possible to estimate the level of the scattered damage accumulation (mainly at the stresses above the OYS level). It was revealed that fractography data, namely the pattern and sizes of the fatigue crack initiation and propagation zones, did not correlate well with the dependences of the parameters of the hysteresis loops.

## 1. Introduction

Methods for estimating an optical flow, i.e., the motion of a brightness pattern observed when relocating an object in front of a camera, or the camera in a stationary environment, have been widely using since the 1980s. These were determined by the capabilities of developed computing systems for high-performance processing of large data arrays. In addition, such approaches began to be utilized for solving R&D problems. As an example, the particle image velocimetry (PIV) method was developed for evaluating velocity fields of liquid or gas flows. In experimental mechanics, the digital image correlation (DIC) method became widespread for assessing strains in solids. Nowadays, it is one of the most common approaches for studying both strain and fracture processes in heterogeneous materials [1,2]. In addition, the DIC method found wide application in solving various scientific and technical problems in materials science [1,3,4], solid mechanics [5], biomedicine [6,7,8], civil engineering [9,10,11,12,13], non-destructive testing (NDT), structural health monitoring (SHM), etc.

For fracture mechanics, the implementation of the DIC method enables to obtain many breakthrough results, since the characteristic scale of strain processes, developing at the tip of a fatigue crack, can be directly observed and numerically analyzed. These data are used for calculating generally accepted quantitative parameters: crack opening displacement (COD), crack tip opening displacement (CTOD), stress intensity factor (CIF), and J-integral [14,15,16]. In this case, metallic materials (alloys) were mostly the objects under study. For instance, the crack propagation patterns were quantitatively investigated for mixed loading modes [17,18,19,20] At the same time, no visible and optically perceived changes could be revealed at the crack initiation stage (i.e., accumulation of scattered damages), even when high microscope magnifications were utilized [21].

Since the DIC technique can be classified as grid one, it was coupled with the finite element method (FEM) [22,23] In addition to improving the accuracy and robustness of estimated displacements and strains, this enabled to verify stain models [24,25,26], especially under the crack propagation [27]. In fracture mechanics, the problem of a discontinuity presence in an optical image (as well as within a strain field) is solved with the use of the extended DIC approach (X-DIC) [28].

Mechanical hysteresis loops, characteristic of cyclic loading, can change their shape and parameters depending on the structural state of the material, which enables to use them for assessing the damage development [29,30,31] The hysteresis loop area [32] and the dynamic moduli [21,33] were estimated by many researchers to analyze processes under cyclic loading. To assess the damages formed during fatigue tests, the reduction in the elastic modulus were mostly used, but not a change in the hysteresis loop areas.

A comprehensive study based on calculation the mechanical hysteresis parameters in the “displacement–load” coordinates was carried out in [34]. These parameters were measured according to readings from strain gauges. It was highlighted in [35] that not only the area, but a change in the shape of the hysteresis loops reflects the development of damage accumulation processes as well. However, some researchers believe that the loop area depends only on cyclic loading frequency, i.e., an ability to develop relaxation processes at high-rate testing [36]. It was stated in [37] that hysteresis related viscoelastic losses include the energy dissipation both on heat generation and on structural changes induced by the cyclic loading.

Most of the models describing the evolution of the hysteresis behavior under cyclic loading were based on phenomenological approaches and were developed for laminated polymer composites. Any mechanisms of the damage accumulation were not considered in these methods, while the corresponding indications (fatigue stiffness, fatigue strength, residual strength, etc.) were adapted to assess their levels. The latter depend on many factors, including loading level, crack propagation mode, fatigue life, cycling frequency, environmental conditions, etc. [38]. It was shown in [39] that neither mechanical nor thermodynamic cyclic stability was observed during fatigue tests. This fact can be caused by non-uniform distribution of strains over the bulk sample, which resulted in corresponding energy dissipation (in the form of hysteresis).

To assess changes in the loop parameters, especially under high-cycle fatigue (HCF), a great measurement accuracy for strain determination is required (it was shown in [40] that non-contact DIC method enables to reach the level of 0.01%). The DIC method was applied to register hysteresis loops in cyclic testing of both neat thermoplastics [39] and thermoset polymers [40]. In addition, a coupling of the DIC and infrared thermography made it possible to assess the strain, as well as levels of dissipated and stored energy. It was shown that the energy dissipated per cycle is always less than the mechanical one, which was related to the area of a hysteresis loop [39]. This difference characterized the contribution of the accumulated energy associated with microstructural changes. Thus, the use of the DIC to assess the parameters of mechanical hysteresis loops is an urgent task that requires the development of appropriate both hardware and software tools, including automation of the cyclic testing and capturing optical images.

This work is aimed at developing a laboratory facility based on a servo-hydraulic testing machine for estimating the parameters of mechanical hysteresis loops by means of the DIC. Then, the developed hardware-software facility was tested by studying the fatigue damage development in a high-performance polymer, i.e., neat PI. It was expected that the parameters of hysteresis loops reflect the polymer damage degree and enabled to characterize its current mechanical state.

The paper is structured as follows. Section 2 provides a description of the materials and methods as well as testing facility; in addition, the grounds for calculating the elastic modulus, the offset yield strength and the parameters of mechanical hysteresis loops by means of the DIC are given. Section 3 and Section 4 present testing results on estimating hysteresis loop parameters as well as the discussion of experimental data on the fatigue behavior of the neat PI. In the final Section 5, the obtained results are summarized as conclusions.

## 2. Materials and Methods

The fatigue tests were carried out of the neat PI fabricated by compression sintering of the “Solver PI-Powder 1600” powder (Solver Polyimide Co., Jiande, China) with an average particle size of 16 µm. The following mechanical properties were determined previously in static tensile tests: ultimate tensile strength (UTS) of 104 ± 3 MPa; offset yield strength (OYS_0.1_) of 45 ± 4 MPa; elongation at break (*ε*) of 6.6 ± 0.5%; the elastic modulus (*E*) of 2.94 ± 0.19 GPa.

“Dog-bone” shape samples were cut with a milling machine. The total length of the samples was 64 mm, the gauge length was 10 mm, and the cross-sectional area was 3.2 × 3.2 mm. The required surface quality was achieved using sandpapers with grits of 1000.

The fatigue tests were carried out according to the ASTM E606 standard under zero-cycle loads at the stress ratio of *R*(σ_min_/σ_max_) = 0. The loading pattern was sinusoidal and the frequency was 1 Hz. Both load and displacement data were registered in real time with a sampling rate of 100 Hz.

**Figure 1:**
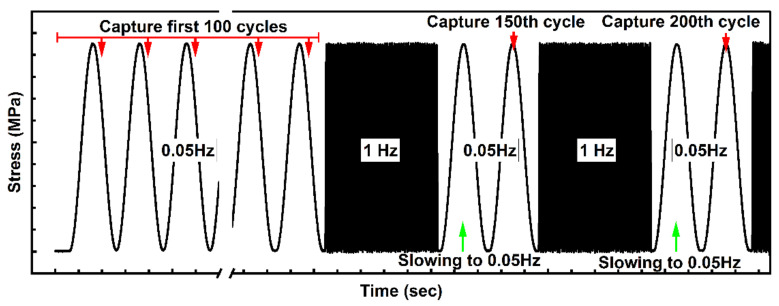
The loading mode with indication of the capturing cycles.

The sample surfaces for the DIC strain measurements were captured for the first 100 loading cycles. Then, images for the loop area calculation were captured at every 50 cycles. The frequency of the tests upon capturing was 0.05 Hz, while it was 1 Hz in other cases. Image capturing frequency was 5 Hz, which enabled to obtain about 100 readings per a loop. The loading modes with indication of capturing cycles are shown in Figure 1.

Engineering stresses were calculated through dividing the force transducer registered load reading over the samples’ cross section (with the latter measured at the onset of cyclic testing).

The fatigue tests were performed at various maximum stresses (σ_max_): 75, 60, 45 and 30 MPa, below and above the Offset Yield Strength (OYS) level. The reason was the need to determine the range of strains, within which assessing of the fatigue properties by the parameters of hysteresis loops was applicable.

Fracture surface were analyzed with the help of scanning electron microscope “LEO EVO 50” (Carl Zeiss, Oberkochen, Germany) at accelerating voltage of 20 kV. Copper films of 10 nm thick were preliminary deposited onto the rupture surface with the help of vacuum evaporator “JEOL JEE-420” (JEOL USA, Inc., Peabody, MA, USA).

### 2.1. The Testing Facility

**Figure 2:**
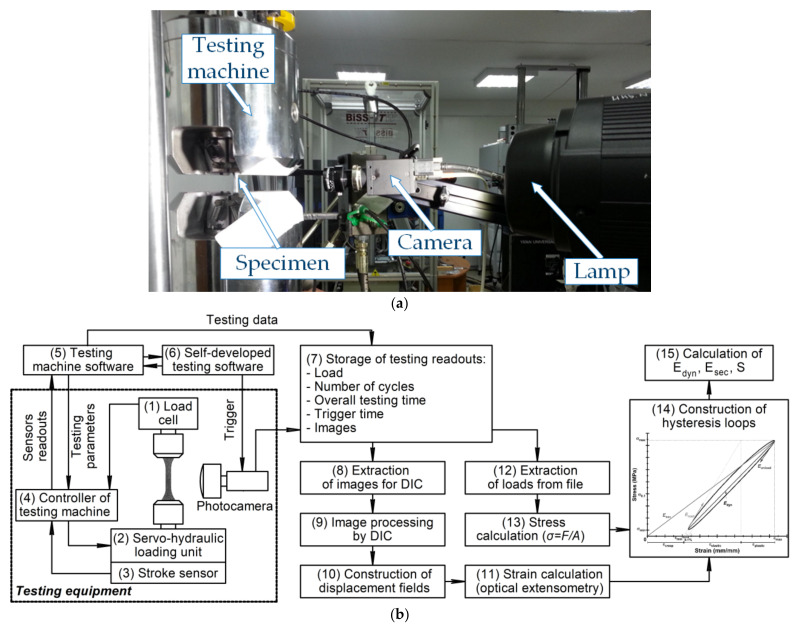
The photograph of testing machine (**a**); the data logging and processing algorithms for the fatigue tests (**b**).

The developed hardware-software facility for fatigue testing consisted of a “Biss Nano” servo-hydraulic machine (Figure 2a) incorporating a “Biss Bi-06-103 15kN” force transducer (Bangalore, India). The latter was installed along the axial direction at the upper part of the frame. Surface images were captured using the “PointGrey Grasshopper 50S5M” digital video camera (Point Gray Research^®^ Inc., Richmond, BC, Canada) with the “Sony^®^ ICX625” 2/3” 2448 × 2048 CCD sensor (a 5 megapixels resolution, an 8.4 × 7.0 mm sensor size with a 3.45 × 3.45 µm pixel dimension). The required level of the surface illumination was provided by a “Jinbei EF-100 LED Sun Light” LED illuminator. A serial port connected controller was used as trigger to control the facility and the camera. The control software was developed by the authors. The schematic of the hardware control and data processing software is presented in Figure 2b.

An alternative strain assessment, being complementary to the DIC, was carried out with a “Bi-06-304” contact extensometer with a gauge length of 12.5 mm (BISS, Bangalore, India).

#### 2.1.1. Strain Calculation by the DIC

Algorithms for displacements measurement over a series of images are generally based on minimizing the functional:

(1) $$\begin{array}{c} \;\;\;\;\;\;\;\;\;\;\;\;\;\;\;\;\;\;\;\;\;\;\;\;\;\;E\left(P,S\right)=E_{D}\left(P,S\right)+\alpha E_{S}\left(P,S\right), \end{array}$$

where P is the displacement function, S is the image region over which the E is minimized, $E_{D}$ is the similarity measure for the image subsets, $E_{S}$ is the similarity measure of vectors in a vector field, α is the regularization factor.

The similarity measure $E_{D}$ of two subsets on a Current Image (CI) and a Reference Image (RI) is determined by various functions via finding their extremum (the minimum, as a rule). The requirements on accuracy, robustness and computation time define a similarity measure complexity. The simplest one is the sum of squared [41]:

(2) $$\begin{array}{c} \;\;\;\;\;\;\;\;\;\;\;\;\;\;\;\;\;\;\;\;\;\;\;\;\;\;\;\;\;SSD=\sum_{X\in S}{\left(F\left(X\right)-G\left(X,P\right)\right)}^{2}, \end{array}$$

where $F\left(X\right)$, $G\left(X,P\right)$ are pixel brightness values in the RI and CI subsets, respectively, X is the pixel coordinate on the plot S, P is the displacement function.

The normalized sum of squared difference with zero mean was used as a similarity measure in the developed algorithm [42,43]:

(3) $$\begin{array}{c} \;\;\;\;\;\;\;\;\;\;\;\;\;\;\;\;\;\;\;\;\;\;\;\;\;\;\;\;\;\;\;\;ZNSSD=\sum_{X\in S}{\left(\frac{F\left(X\right)-\bar{F}}{\sqrt{\sum_{X\in S}{\left(F\left(X\right)-\bar{F}\right)}^{2}}}-\frac{G\left(X,P\right)-\bar{G}}{\sqrt{\sum_{X\in S}{\left(G\left(X,P\right)-\bar{G}\right)}^{2}}}\right)}^{2}, \end{array}$$

where $\bar{F}$ and $\bar{G}$ are the arithmetic mean values of the RI and CI subsets, respectively.

To determine the minimum of the functional *E*, two approaches were implemented:

1. The block method. It is based on determining the position of the *S* region within a reference image when scanning over the second (current) one within a sliding window. Usually, the method utilizes the fast Fourier transform (FFT). This approach is used for a preliminary search for large amplitude displacements. The method is employed neither when the displacement function is complex nor at sub-pixel accuracy.
2. The differential method. In the studied case, the Newton-Raphson numerical optimization algorithm was implemented for calculations with the subpixel accuracy, since it provides stable convergence in the [−1, +1] pixel range.

Optimization by the Newton-Raphson method [44,45] was applied as follows:

(4) $$\begin{array}{c} \;\;\;\;\;\;\;\;\;\;\;\;\;\;\;\;\;\;\;\;\;\;\;\;\;\;\;P^{k+1}=P^{k}-\frac{\nabla E\left(P^{k}\right)}{\nabla \nabla E\left(P^{k}\right)},\;P=\left[p_{0}\ldots p_{5}\right], \end{array}$$

where *k* is the iteration number, *P* is the parametric vector containing the displacement function factors.

The system of equations describing displacements in the region *S* is based on the affine function and has the following form [1,46],

(5) $$\begin{array}{c} \;\;\;\;\;\;\;\;\;\;\;\;\;\;\;\;\;\;\;\;\;\;\;\;\;\;\left\{\begin{array}{c} \tilde{x}\left(x,y\right)=p_{0}+p_{2}x+p_{4}y\\ \tilde{y}\left(x,y\right)=p_{1}+p_{3}x+p_{5}y \end{array}\right. \end{array}$$

The developed installation was implemented for both two- and three-dimensional cases. When operating in the 3D-mode, processing of stereo pairs of images is required in order to determine spatial coordinates of the object’s surface points. Preliminary, calibration is carried out for the stationary located 3D-system to assess parameters of individual cameras and the system as a whole. The parameters contain (i) matrices of projective transformations of the object on the camera plane; (ii) distance; (iii) angles between the camera planes, etc. Restoration of three-dimensional coordinates for the stereopair points is accomplished by calculating the disparity map, which is the displacement of image points from the right camera relative to the left one. The disparity maps are considered using the above (correlation) algorithms. The obtained spatial coordinates of the points are recalculated into displacements, whose totality forms a spatial field of displacement vectors.

Two algorithms were implemented to estimate strains:

- evaluation of the specimen elongation in regard to two reference points (an optical extensometer);
- the total strain field calculation in the region of interest (RoI).

#### 2.1.2. The Optical Extensometry

The sample elongation between two points p1 and p2 is defined as the difference between two Euclidean distances before and after loading. The strain is calculated as follows:

(6) $$\begin{array}{c} \begin{array}{c} \varepsilon =\frac{L_{i}-L_{0}}{L_{0}},\\ \;\;\;\;\;\;\;\;\;\;\;\;\;\;\;\;\;\;\;\;\;\;\;\;\;\;\;\;\;\;\;\;\;\;\;\;\;\;\;\;\;\;\;\;\;\;\;\;\;\;\;\;\;L_{0}=\sqrt{{\left(x_{2}-x_{1}\right)}^{2}+{\left(y_{2}-y_{1}\right)}^{2}+{\left(z_{2}-z_{1}\right)}^{2}},\\ \;\;\;\;\;\;\;\;\;\;\;\;\;\;\;\;\;\;\;\;\;\;\;\;\;\;\;\;\;\;\;\;\;\;\;\;\;\;\;\;\;\;\;\;\;\;\;\;\;\;\;\;\;L_{i}=\sqrt{{\left(x_{2}^{’}-x_{1}^{’}\right)}^{2}+{\left(y_{2}^{’}-y_{1}^{’}\right)}^{2}+{\left(z_{2}^{’}-z_{1}^{’}\right)}^{2}}, \end{array} \end{array}$$

where ($x_{1},y_{1},z_{1}$), ($x_{2},y_{2},z_{2}$) are coordinates of extensometer (reference) points before loading, ($x_{1}^{’},y_{1}^{’},z_{1}^{’}$), ($x_{2}^{’},y_{2}^{’},z_{2}^{’}$) are ones of the same points after loading increment.

#### 2.1.3. The Algorithm for Selecting the RoI and the Coordinates of the Extensometer Points

**Figure 3:**
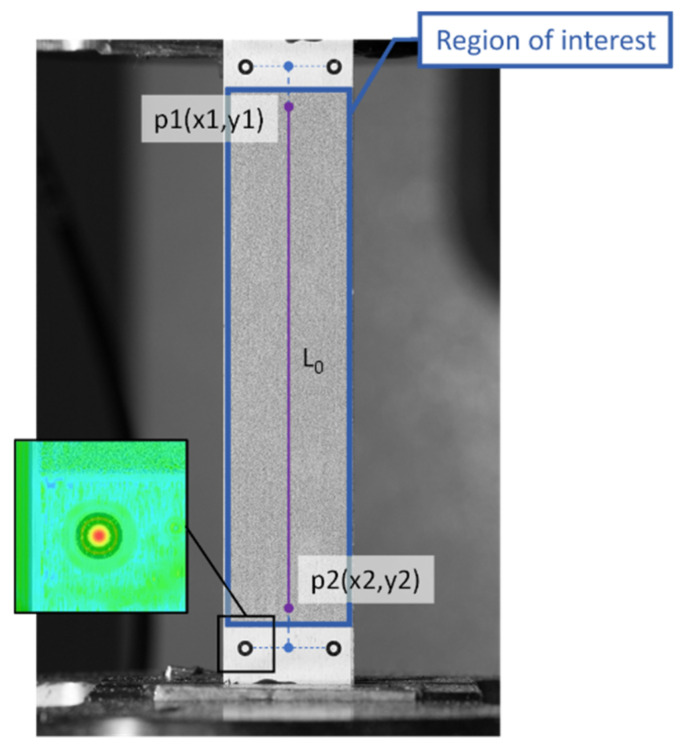
The response function of the mark detection algorithm and the relative position of the extensometer points p1 and p2.

In the case of calculating the full strain field, the selection of a RoI is the preliminary stage (Figure 3).

**Figure 4:**
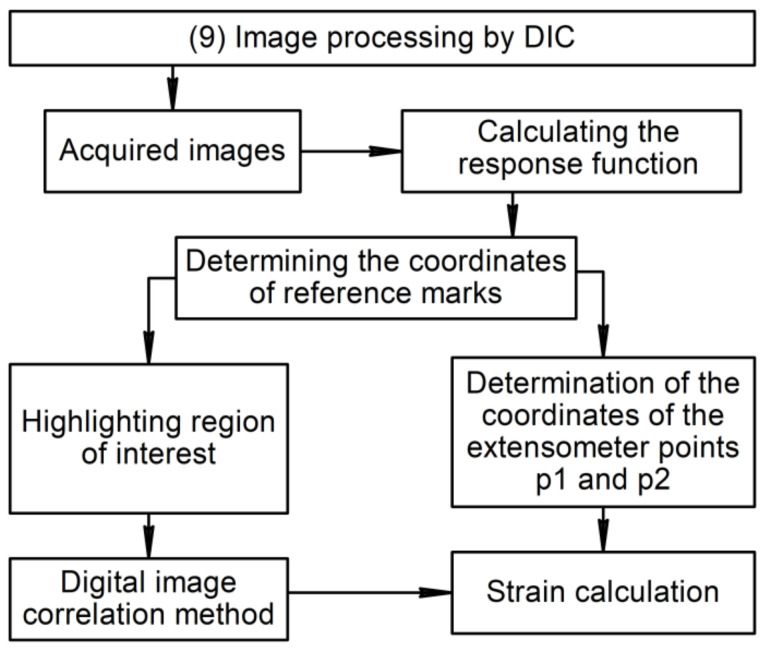
Schematic for the Optical Extensometer operating principle.

As such, there is an image portion containing the gauge length of the test sample, as a rule. This procedure should be performed automatically. The schematic of the algorithm for image processing by DIC is illustrated in Figure 4. To do this, reference marks are highlighted on the sample surface. In the studied case, speckle patterns and the marks were deposited using an inkjet printer with ultraviolet curable ink (UV printing) at a 600-dpi resolution. A correlation-based mark detection algorithm was developed. When marks are detected, it becomes possible to determine the RoI and the extensometer points.

#### 2.1.4. The Calculating the Loop Parameters

**Figure 5:**
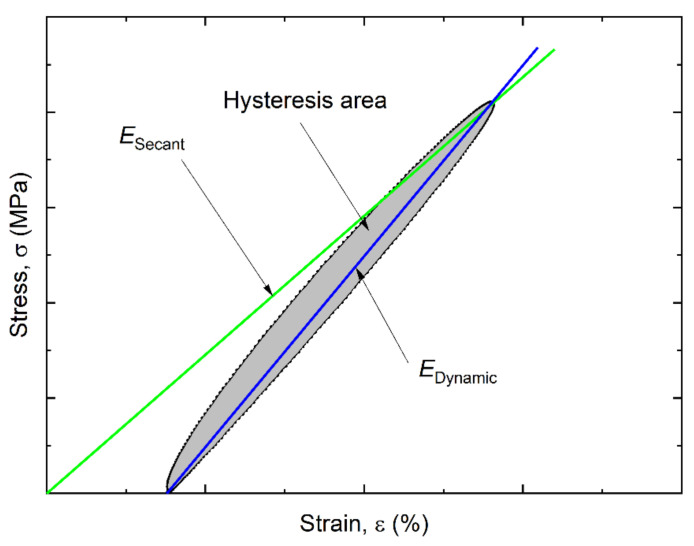
Parameters of the mechanical hysteresis loop.

In this research, the following loop parameters were determined: (i) area, (ii) secant modulus, and (iii) dynamic modulus, Figure 5. The load was measured with the use of force transducer (gauge) of the Biss testing machine. The stress was calculated by dividing the measured force over the cross-section area.

The loop area was calculated as the difference between integrals of two loop parts, reflecting both loading and unloading half-periods (half-cycles), respectively:

(7) $$\begin{array}{c} \;\;\;\;\;\;\;\;\;\;\;\;\;\;\;\;\;\;\;\;\;\;\;\;\;\;\;\;S=\overset{\varepsilon_{max}}{\underset{\varepsilon_{0}}{\int }}\sigma \left(\varepsilon \right)d\varepsilon -\overset{\varepsilon_{1}}{\underset{\varepsilon_{max}}{\int }}\sigma \left(\varepsilon \right)d\varepsilon \end{array}$$

where $\varepsilon_{0}$, $\varepsilon_{1}$ are the strain levels corresponding to the beginning and end of a cycle.

The dynamic and secant moduli of elasticity were determined as follows:

(8) $$\begin{array}{c} \;\;\;\;\;\;\;\;\;\;\;\;\;\;\;\;\;\;\;\;\;\;\;\;\;\;\;\;\;E_{dyn}=\frac{\sigma_{0}-\sigma \left(\varepsilon_{max}\right)}{\varepsilon_{0}-\varepsilon_{max}};E_{sec}=\frac{\sigma \left(\varepsilon_{max}\right)}{\varepsilon_{max}} \end{array}$$

## 3. Results

### 3.1. Trial Test Results of the DIC-Based Calculation of the Loop Parameters

When using the optical strain measurement method, an important aspect was its synchronization with data readings of the force transducer of the testing facility. Processing of raw data did not enable to accurately determine both shape and parameters of hysteresis loops due to high noise level. Therefore, a trial experiment was performed in order to identify and subsequently minimize its influence. In this case, both the contact extensometer and the non-contact DIC method were utilized simultaneously to assess strains.

**Figure 6:**
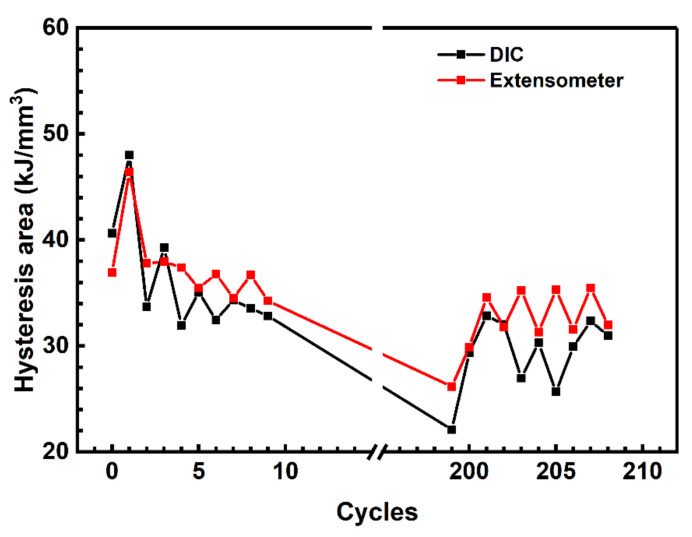
The results of the trial experiment on the calculation of the loop areas from the contact extensometer and the remote DIC method.

The facility was tested by evaluating the parameters of hysteresis loops during the trial cyclic tests in the load control mode. Figure 6 shows plots of the loop area variation based on the data from the contact extensometer as well as DIC ones. This suggests that both methods gave comparable results, which enabled to use the developed DIC-based system to determine strains as well as construct hysteresis loops. The experiments were conducted 5 times. The scatter did not exceed 10%.

An important factor affecting the results of calculating the loop parameters was the time spent on registering one loop (which had to be minimal for lowering the influence of relaxation processes). Note that the system was limited by the camera image capturing rate (frequency of 5 Hz). On the one hand, the number of registered images could be reduced. In this case, a sample was loaded at a frequency of 0.2 Hz, which was comparable with standard test conditions (1 Hz). On the other hand, rising the number of images by 10 times, i.e., up to 250, could improve the calculation accuracy of the loop parameters. However, the loading duration enhanced up to 50 s in this case, which could be accompanied by the development of relaxation processes that were not typical for the implemented cyclic loading mode (0.02 Hz versus 1 Hz). For this reason, another trial experiment was conducted to compare the data for the loops constructed over 25 and 250 images (at the loading frequencies of 0.2 and 0.02 Hz, respectively). The loading data recorded using the testing facility software embedded could be distorted due to noise and interference. In order to reduce their influence, a software approximation of the loading data was carried out. Since the sinusoidal loading pattern was utilized during the cyclic tests, a sine-type function was also applied as an approximating one:

(9) $$\begin{array}{c} \;\;\;\;\;\;\;\;\;\;\;\;\;\;\;\;\;\;\;\;\;\;\;\;\;\;\;\;\;\;\;\sigma \left(t\right)=m+A\sin\left(\omega t+\varphi \right), \end{array}$$

where m is the average value of the loading function, A was the loading amplitude, ω and φ are frequency and phase, respectively. The strain values obtained using the DIC were also approximated.

**Figure 7:**
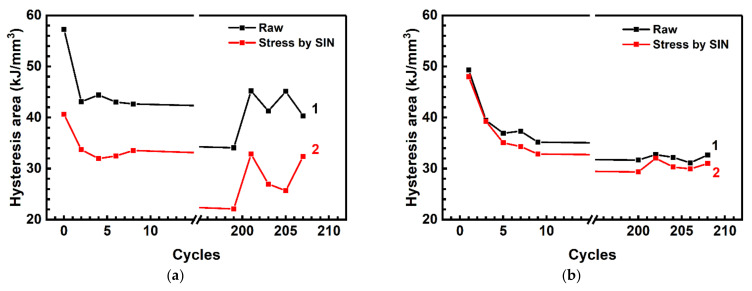
Changes in the loop area for the raw (1) and approximated (2) data calculated over 25 (**a**) and 250 (**b**) images.

The test was performed at the loading frequency of 1 Hz with data capturing at the first 20 cycles. Then, the cycling stage followed without any captures. Starting from the 200th cycle, the next 20 ones (with capturing) were carried out. In doing so, loops containing 25 and 250 points were alternately recorded. Figure 7 shows the area values calculated using those data. 

In the case of a rare sampling (25 points per loop), the approximation slightly changed the curve pattern. Pronounced fluctuations were found as the number of cycles enhanced (Figure 7a). With the frequent sampling of 250 points (Figure 7b), the graph was significantly smoother, regardless of the applied approximation. In addition, a dependence of the loop area on the loading rate was constructed. The rising of the latter caused a greater increase in the loop area. With a smaller number of points (25) for constructing the loops, “underestimated” values were obtained. This resulted from the approximation, while the loop area values were almost at the same level as the raw data were processed over 250 points per loop (Figure 7b). In Figure 7a, an abrupt increase in the Energy loss parameter was evident in the region of *n* = 200 cycles, which suggested to be due to a sharp change in the loading rate from 1.00 down to 0.02 Hz.

Thus, slowing down the loading rate increased the measuring accuracy for the loop parameters. On the other hand, the strain response changed due to the development of relaxation processes. In addition, one should avoid capturing images immediately after changing the loading rate. The above-described results enabled to recommend the following testing parameters: the number of points per loop of 100 and the loading rate of 0.05 Hz. In addition, the “slow” preliminary loading cycle at 0.05 Hz is recommended to be applied before image capturing for loop constructing.

**Figure 8:**
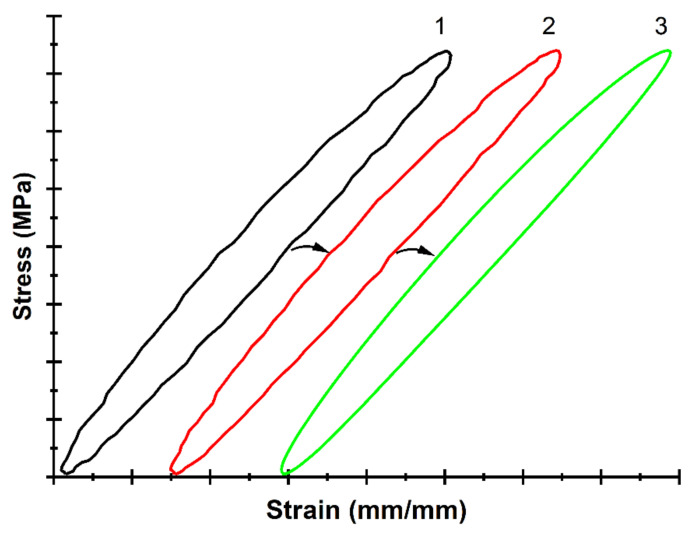
The hysteresis loops constructed at the 100th loading cycle using the raw data (1), after the approximation by the sin function (2), as well as after the simultaneous approximation of stresses by the SIN function and strains by the B-spline one (3).

Figure 8 (loop #1) shows mechanical hysteresis loops for neat PI obtained after running 100 loading cycles (when the strain response of the material to the load became steady). The loop constructed using the raw data was characterized by a non-smooth variation due to the noisy stress-strain readings (Figure 8). This resulted in raising an error in the calculation results of both the elastic modulus and the loop area. It was proposed to approximate the raw data on stresses (loads) using the SIN function, since the applied load was sinusoidal as well.

The influence of the approximation on the loop shapes is illustrated by Figure 8 (loop #2). However, such a function could be applied for the raw DIC data approximation at low loads only. When exceeding the elastic limit, the nonlinear “strains–stresses” dependence caused its distortion from the sinusoidal pattern. Therefore, the B-spline function was applied to approximate the raw data.

The strain values were approximated by the global B-spline function using the SciPy library [47]. After determining the approximated strain function *ε*(*t*), its extremum *ε*_max_ was estimated. The level of the latter was required to calculate the loop parameters. The use of simultaneous approximations of the stresses and strains showed efficiency, since a smooth hysteresis loop of a regular shape was constructed with almost no distortion (Figure 8, loop #3).

The above results have proven the efficiency of the DIC for hysteresis loop constructing. The rational parameters for calculations were determined within the framework of the trial experiment. Further, a comparative study on the fatigue failure of neat PI under various stress amplitude was carried out using the developed method (in terms of the parameters of mechanical hysteresis loops).

### 3.2. Fatigue Testing

**Figure 9:**
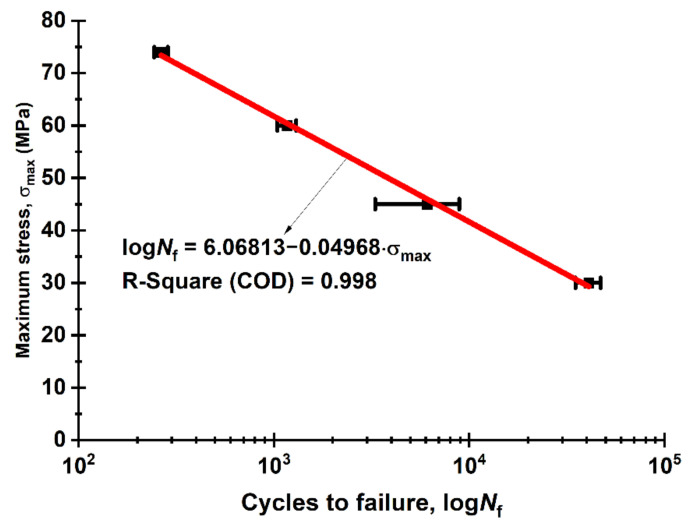
The fatigue curve for the neat PI (red line) with the linear fit.

Figure 9 shows the “maximum stresses vs. the number of cycles to failure” fatigue (Wöhler) curve for the neat PI in the semi-logarithmic coordinates. It showed the linear changing nature in the entire studied range. Most likely, this was due to the fact that the fatigue failure mode transformed from the ultra-low-cycle type to the low-cycle one (*N*_p_ < 10^5^ cycles). At least five specimens of each stress level were tested.

**Figure 10:**
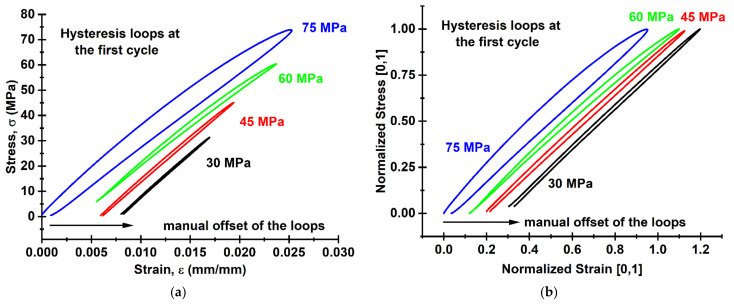
The hysteresis loops for the first test cycle at the maximum stresses (σ_max_) of 30, 45 and 60 and 75 MPa. Engineering stress-strain diagram (**a**); normalized stress-strain diagram (**b**).

Then, the parameters of the mechanical hysteresis loops were analyzed for the applied modes of the cyclic tests. Figure 10 presents ones recorded during the first test cycle at different load levels. The loops in the plot were manually shifted to the right in order to clearly observe the shape of each curve. With its rising, the loop width values enhanced as well. This result was expected, since a higher load level implied an increase in the proportion of inelastic material strain, and vice versa.

**Figure 11:**
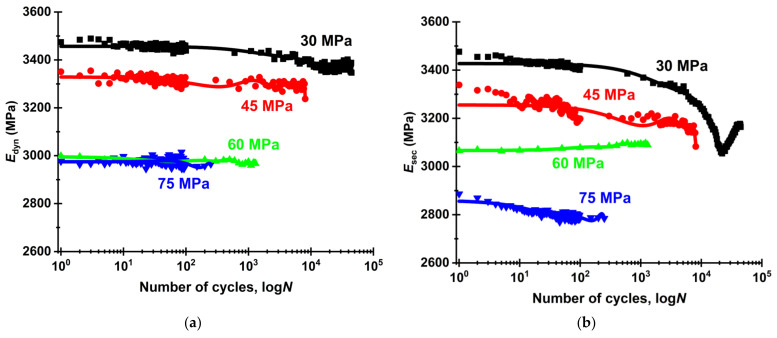
The dependences of the dynamic (**a**) and secant (**b**) moduli on the number of cycles at different maximum stresses (σ_max_).

Figure 11 shows dependences of the secant and dynamic moduli versus the number of cycles. In general, the values of both ones increased with decreasing the load level. The secant modulus (*E*_sec_) remained constant and even slightly rose at the load of 60 MPa and, conversely, had a clear downward trend at 45 and 30 MPa. This lowering dynamic was expressed as shifts of the loops to the right along the strain axis and reflected the development of residual irreversible strains in the material. Thus, residual strains were observed at the load values of 45 and 30 MPa, but were not evident at 60 and 75 MPa. This indicated their different nature at the load levels below and above the OYS level. The authors suggest that strains (including plastic ones) developed over the entire sample bulk at 60 and 75 MPa, while the process localized in regions only with the greatest stress concentrations at 45 and 30 MPa, which did not cover the entire sample volumes.

At the load level of 30 MPa, the *E*_sec_ value reduced closer to the moment of failure. The authors attributed this fact to the local damage accumulation. At 45 MPa, a decrease in the analyzed parameter was also observed at an approximately constant rate throughout the entire test. At 60 and 75 MPa, the *E*_sec_ value almost did not change, and failure occurred with a significant proportion of plastic strains, but not due to the local damage accumulation in this case.

**Figure 12:**
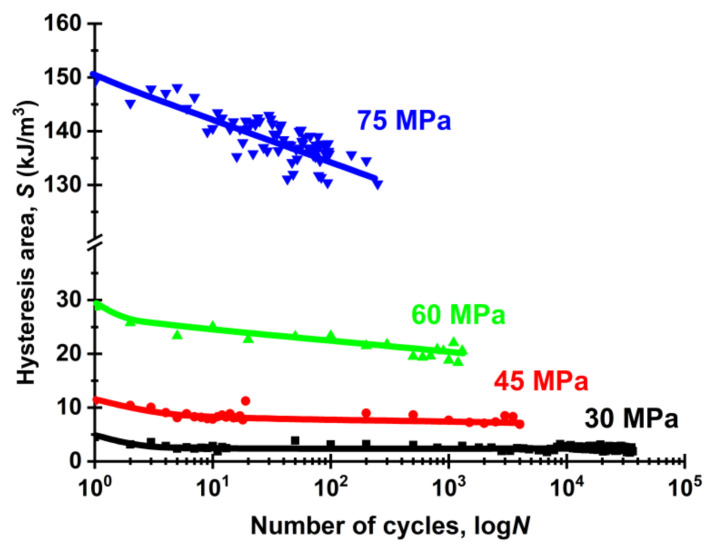
The dependences of the loop areas (*S*) versus the number of cycles to failure at different maximum stresses (σ_max_).

Figure 12 shows graphs of the loop area (S) dynamics during the fatigue tests at the applied load levels. In all cases, the S values reduced linearly: from 6.5 down to 3.5 kJ/m^3^ (for the first and last test cycles, respectively) at 30 MPa, from 18.7 down to 9.9 kJ/m^3^ at 45 MPa, from 27.2 to 21.2 kJ/m^3^ at 60 MPa and from 150.2 to 132.1 kJ/m^3^ at 75 MPa. The results of the analysis of the changes in the S parameter during the cyclic tests showed that the rate of its decrease was mostly constant (linear) till failure. In so doing, there was no region of stable values at the highest load levels of 60 and 75 MPa. At 45 MPa, the area of stable values was reached after about 10 cycles. At 30 MPa, which actually corresponded solely to elastic strains, the S parameter only slightly reduced in the initial test stage. Thereby, it was sensitive to load-depended structural transformations.

**Figure 13:**
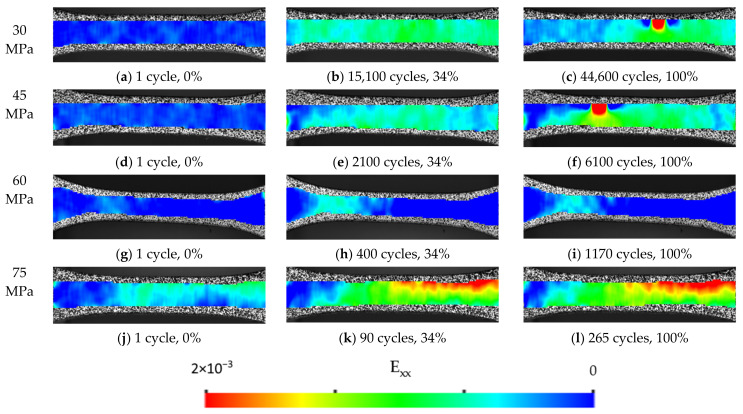
Strain fields calculated for neat PI at different levels of maximum load in the cycle (σ_max_): 30 MPa—(**a**–**c**); 45 MPa—(**d**–**f**); 60 MPa—(**g**–**i**); 75 MPa—(**j**–**l**). The value of cyclic operating time: 0%—(**a**,**d**,**g**,**j**); 34%—(**b**,**e**,**h**,**k**); 100%—(**c**,**f**,**i**,**l**).

DIC was employed as an optical extensometer for non-contact calculation of mechanical characteristics and parameters of hysteresis loops; however, this method makes it possible to calculate 2D strain fields on the sample surface. The evolution of deformation fields for different loading amplitudes and different number of cycles is shown in Figure 13. Note that the fields were calculated only for images registered at the maximum load in each cycle. The images acquired at maximum load on the first cycle were set as Reference ones. This approach allows one to analyze both the absolute stain value as well as to evaluate the changes in the deformation distribution during cyclic loading. This substantially increased the sensitivity of the technique to damage nucleation and deformation localization development.

The left column illustrates the fields registered at the second loading cycle; the middle column corresponds to 34% of the number of cycles prior failure; the right one shows the stain fields at the last photographic cycle prior to failure. It is seen that the deformation fields are different at various levels of the maximum load in the cycle.

For σ_max_ = 30 MPa, the strain level was low and not localized at the first few loading cycles. However, after ~13,000 cycles, an increase in the strain level was observed. It was localized near the lower grip and then developed over the entire gauge length of the sample during the next 2000 cycles (Figure 13b). This process was evident both in the deformation fields, as well as at the *E*_sec_ and *E*_dyn_ graphs (at the same number of cycles, Figure 11). At ~2,000 cycles before fracture, a region of pronounced strain localization was formed in the middle part of the sample (Figure 13c). An increase in its value was accompanied by the decrease of the one in the surrounding material. This indicated the development of a crucial defect and pointed out the region of final failure occurrence.

A similar behavior was observed at a higher level of cyclic load (namely 45 MPa, Figure 13d–f). Obviously, the duration of each stage took fewer cycles, in this case.

For loads above the yield point (OYS _0.1_ = 45 ± 4 MPa), an increased level of strain was revealed from the very onset of testing (Figure 13g,j). Its level increased rather quickly (Figure 13h,k). However, the sample failed due to plastic deformation (drawing) rather than from fatigue crack growth (Figure 13i,l). The stage of crack propagation was short (in terms of the number of cycles), which resulted in a quick final fracture.

The use of the 2D–DIC made it possible to register changes in the parameters of hysteresis loops as well as strain fields for this stage of fatigue failure due to its long duration. On the other hand, at high levels of cyclic loading (of 60–75 MPa, that is above the yield point), the final fracture stage associated with the propagation of a macrodefect was extremely short. Due to the chosen photographic interval, it was not possible to reveal these processes on the strain fields calculated by means of the DIC.

**Figure 14:**
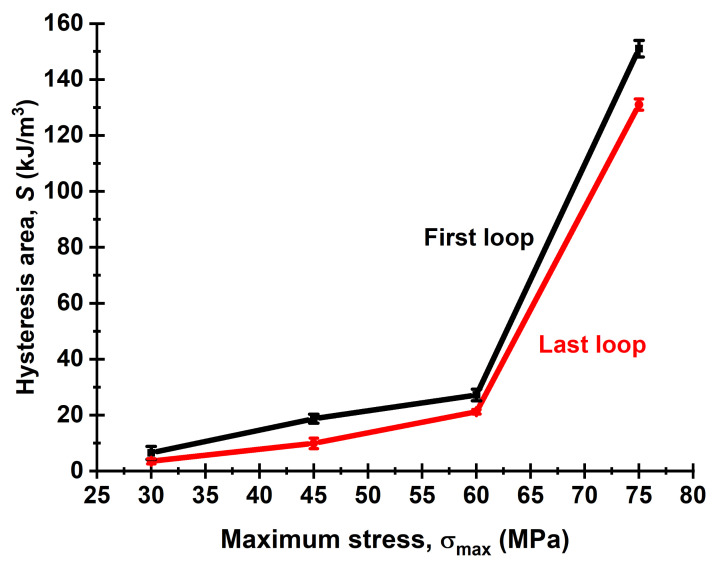
The dependences of the loop areas (S) on the maximum stresses (σ_max_): 1—for the first cycle; 2—for the cycle preceding failure.

Figure 14 presents dependences of the areas of hysteresis loops (S) for the first cycle and the one preceding failure from the maximum stress levels. Both of them were characterized by nonlinear changes in the studied range. However, by the moments of the sample failure, the decreases in the S values were about 5 kJ/m^3^ for the range 30–60 MPa, while it exceeded 10 kJ/m^3^ for the cycling stress of 75 MPa. Therefore, the stress of 60 MPa should be considered as the threshold one. Above this stress the fracture developed under ultra-low cycle fatigue mode, which was accompanied by a continuous rearrangement of the polymer microstructure with application of cyclic loads.

**Figure 15:**
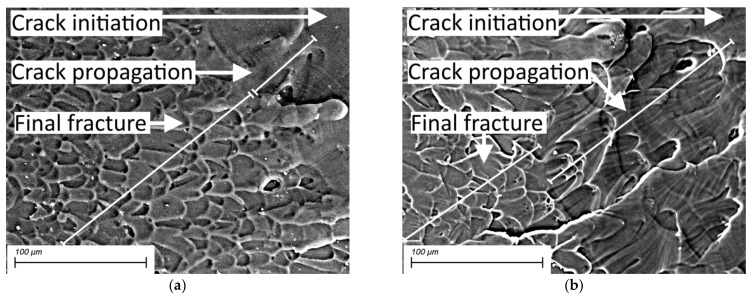
The SEM-micrographs of the neat PI fracture surfaces after the fatigue tests at 60 MPa (**a**) and 30 MPa (**b**).

As noted above, the crack propagation stage was extremely short for the neat PI regardless of the load level. To elucidate the effect of the maximum stress values, SEM-micrographs (Figure 15) of the neat PI fracture surfaces after the fatigue tests at 30 and 60 MPa (below and above the OYS, respectively) were analyzed. Their key difference could be reported as the following one. At the stress value of 60 MP (above the OYS level), there was actually no crack propagation zone, typically characterized by a radial striated relief (Figure 15a). At the same time, pronounced bands of the fatigue crack propagation were observed at the load level below the OYS point (Figure 15b). Thus, the results of the fractographic studies, namely the pattern and sizes of the fatigue crack initiation and propagation zones, did not directly correlate with the dependences of the parameters of the hysteresis loops.

## 4. Discussion

In the first experimental part of the paper a comparison of strain measurement results, obtained with the use of the DIC in contrast with a contact extensometer registered ones were carried out (see the Section 3.1). The area of hysteresis loops was calculated with the use of both methods. Note that the saw-tooth shape of the graphs (Figure 6) was related to the alternation of testing cycles with different loading frequency of 0.2 Hz and 0.02 Hz. It gave rise to a slightly different behavior of the polymer. For this reason, different responses of the measuring methods were attained. This illustrates the sensitivity of the proposed technique. In addition, the hysteresis nature of polymers’ fatigue was proven that correlates well with the literature data.

In addition, it was shown that the DIC underestimates the area of the loops due to the low frequency of image capturing. However, the data of both contact extensometer and DIC are close at a high imaging rate. In doing so, the appropriate sampling rate of 100 images (points) per loop has been concluded to provide a precise measurement accuracy. This is of importance for developing the system and planning fatigue tests. The obtained results correlated well with the readings from the contact extensometer. Thus, the results presented in Section 3.1 verified the reliability of the method used. In addition, the consistency of the developed DIC system measurements was proven in other authors’ investigations, where the results were compared both with a contact extensometer as well as the data obtained with the use of commercial DIC systems, for example, VIC-2D (Correlated Solutions, Irmo, SC, USA).

The rest of the experimental studies (Section 3.2) was conducted with the use of commercially available testing machine at exactly specified testing condition and parameters. They might be reproduced elsewhere. The parameters of hysteresis loops were calculated according to referenced approach. Their sensitivity to testing parameters was illustrated. The test coupons were fabricated according to the corresponding ASTM recommendations. The number of specimens were at least 5 for each testing conditions. The scatter bars are shown on the graphs. We suggest that the data presented as well as conclusions made are reliable.

It should be stressed that fatigue testing results are very condition sensitive: first of all, on the polymer structure (that depends greatly on sintering conditions) as well as fatigue testing mode (at stress or strain control, for instance) and loading parameters. Some of testing results were not included in the manuscript regardless the were taken into account at designing the system and planning the experiments.

As a discussion of the obtained results, the authors proposed their following interpretation. The decrease in the S parameter with rising the number cycles was associated with the gradual localization of strains due to the scattered damage development. In the initial cycles, the entire samples were deformed quasi-uniformly. The hysteresis loops were, respectively, determined by enhancing of an internal friction of polymer macromolecules, when the entire volumes of the loaded samples were involved in their resistance to stresses. It is known that the polymer structure was characterized by a random and “entangled” arrangement of macromolecules, which caused local overstresses under the loads (in favorably and unfavorably oriented ones relative to the loading axis). These local overstresses led to the development of localized plastic strains and breaking fragments of macromolecular chains. In turn, this contributed to the further concentration of stresses in such zones, and new regions of the macromolecular structure were gradually involved in the damage development process.

The formation of local stress concentrators caused the strain inhomogeneity, where some local areas were subjected to significant deformations, but the rest of the samples, on the contrary, were deformed to a lesser extent. As a result, with a decrease in the volume of macromolecules not involved in the process, the internal friction also reduced, which was reflected in the form of the hysteresis loops. In other words, the decrease in the loop area reflected the polymer strain degree. It was possible to assess the accumulated damage level and to predict the residual life by estimating the S parameter value (mainly under the cyclic loads above the OYS level).

Since the mechanical hysteresis loop characterizes the viscous-plastic behavior of polymers, its parameters can contain important information about the nature of the response of a cyclically loaded polymer. The latter depends on its current mechanical state. The hysteresis in metals is mostly associated with crack opening and closing, while the stage of crack growth in high-strength (thermoplastic) polymers is extremely short. Therefore, the rearrangement of macromolecules (at the initial stages of loading) and the development and consolidation of damage at the final stages are responsible for the development of this process [48]. Traditionally, the parameters of the hysteresis loops are estimated from the testing machine stroke displacement, since the contact strain gauge sensor cannot be used due to the rapid brittle failure. In this regard, the partial aim of this paper was to develop an approach to non-contact measurement of strain.

On the whole, the results obtained are in good agreement with the literature data. For instance, McKeen [49] reported that the hysteresis loop shape and area stabilized upon 10% of the total life time and remained constant after. In addition, the loss of viscoelastic energy due to the hysteresis included energy dissipation for heat generation and structural changes upon cyclic loading. Decreasing of the hysteresis loop area under fatigue was revealed in PI-based composites tested in tension mode [50]. In contrast, loops with increasing area were found for these composites tested in alternative mode. Thus, hysteresis loop area is an important parameter that makes it possible to study both linear and nonlinear behaviors of the particulate composites, as well as to assess their damage degree under cyclic loading [51,52].

Pastukhov [53] showed that plastic deformation dominates failure development at high levels of applied stresses. In doing so, at short loading time the applied load promotes the plastic flow of the polymer matrix, while its accumulation upon reaching the critical level gave rise to fracture. At longer loading times and lower applied stresses, cyclic loading accelerates crack growth. In this concern, machining defects within the material initiate cracks that propagate under the applied load until one of the cracks reaches a critical size and failure occurs.

Authors of this study conducted similar experiments of hysteresis loop parameters for another high-performance polymer PEEK [54]. It was shown that the parameters of the analyzed parameters of hysteresis loops in both low- and high-cycle fatigue modes also differ since the viscous-plastic properties of PEEK and PI are noticeably different.

In order to summarize the outcomes of the research, the following is to be concluded. An automated experimental setup has been developed that includes synchronized completely automated hydraulic testing machine and remote strain measurements. The installation was adjusted for fatigue testing of high-performance polymer and their composites, with compensation for noise and other signal disturbances. A series of experiments were performed for neat PI samples when testing conditions (stress amplitude) were varied. It was shown that manifestation of the hysteresis at fatigue of polymers (composites) might be revealed and numerically estimated with the use of the designed automated system. In the forthcoming paper, the obtained results will be used for numerical FEM study of the PI-based composites fatigue with the use of the secant modulus and loop area as a fatigue life sensitive parameter.

An additional advantage of the developed system is to be outlined, regardless, it was not the matter of the study. The 2D DIC version of the system makes it is possible to conduct local measurements, i.e., in different location of test coupons. This is of crucial importance when notched samples are tested. Moreover, during running the testing, this data is of importance, in order to ensure (track) uniformity of loading in various directions as well as to reveal any heterogeneity in strain development.

## 5. Conclusions

Based on the servo-hydraulic testing machine, whose control was synchronized with the DIC-based strain measurement optical system, the laboratory facility was designed. It allows to study the fatigue processes of the high-performance polymers using the parameters of the mechanical hysteresis loops. The stress data logging was based on the hardware readings from the force transducer. Strain assessment was carried out by the DIC method, which improved the accuracy by measuring only within the gauge length (with a high localization degree). Strains were estimated in two ways: (a) in the optical extensometer mode; (b) via calculation of the total strain field in the region of interest (RoI).

In order to reduce the influence of noise and interference, the software approximation of the stress–strain data was carried out. It was shown that the use of the sine function as an approximation one was effective at loads below the yield point. The strain values calculated from the data, obtained by the DIC, were efficiently fitted by the global B-spline function.

According to the registered data on stresses and strains, the mechanical hysteresis loops were constructed, with the subsequent determination of their areas and moduli. The latter were assessed using the slope of the straight line connecting the points of the minimum and maximum strains (the start and end loading moments). This parameter was a complex value reflecting the viscous and elastic strain components. For the fatigue test of the PI, reducing the loading frequency down to 0.02 Hz and increasing the number of points per loop up to 250 resulted in the smoother change in the loop area (energy loss).

The changes in the areas of the mechanical hysteresis loops, as well as both secant and dynamic moduli were analyzed. These parameters enabled to differentiate the neat PI behavior at different levels of the cyclic loads. It was found that the damage accumulation was accompanied by the decrease in the loop areas, and failure occurred when it reduced by at least ~5 kJ/m^3^. The secant modulus remained nearly constant at the loads of 75 and 60 MPa, while, on the contrary, it showed the clear downward trend at 45 and 30 MPa. This indicated different strain and fracture mechanisms under the cyclic loads below and above the OYS level. Thus, lowering the loop area along with changing the secant modulus value made it possible to estimate the level of the scattered damage accumulation and to assess the residual service life of the cyclically loaded polymer mainly at the stresses above the OYS level.

At low cycling loads of 30–45 MPa of neat PI, the fatigue failure was accompanied by a pronounced stage of fatigue crack propagation (that was proven by the SEM micrographs). The use of the 2D DIC made it possible to register changes in the parameters of hysteresis loops as well as strain fields for this stage of fatigue failure due to its long duration. On the other hand, at high levels of cyclic loading (of 60–75 MPa, that is above the yield point), the final stage associated with the propagation of a macrodefect was extremely short. Due to the chosen photographic interval, it was not possible to reveal these processes on the deformation fields calculated by means of the DIC.

It was shown that the estimation of mechanical hysteresis loops in terms of their area, as well as the secant and dynamic moduli, obtained by the DIC can be employed to interpret the difference in the fatigue characteristics at the stage of scattered damage accumulation. However, this does not enable to unambiguously predict residual life. The solution of this problem requires systematic research using the approaches of fracture mechanics and attracting highly sensitive means of nondestructive testing.

## Data Availability

Not applicable.
